# Gender Variations in Alveolar Process Dimensions and Maxillary Anterior Tooth Inclination: A CBCT‐Based Cross‐Sectional Study

**DOI:** 10.1002/cre2.70508

**Published:** 2026-11-09

**Authors:** Rajashri A. Kolte, Abhay P. Kolte, Vinisha A. Bajaj, Ritika R. Gattani, Dharmarajan Gopalakrishnan

**Affiliations:** ^1^ Department of Periodontics and Implantology Ranjeet Deshmukh Dental College & Research Centre, Lata Mangeshkar University Nagpur, Digdoh Hills India; ^2^ Department of Periodontology Manipal College of Dental Sciences, Manipal Academy of Higher Education Manipal India

**Keywords:** alveolar bone, anterior maxilla, cone beam computed tomography

## Abstract

**Objectives:**

Due to the unique anatomy of the maxillary anterior esthetic zone, placing an implant immediately post‐tooth extraction can be challenging. The success of immediate implant placement largely relies on the shape of the alveolar bone and the angle of the extracted tooth. This cross‐sectional study is designed to evaluate the dimensions of the alveolar bone in the maxillary anterior region using cone beam computed tomography (CBCT).

**Methods and Material:**

Maxillary anterior region scans from 60 individuals (30 males, 30 females) were assessed using CBCT. Statistical analysis accounted for the clustering of teeth within individual participants. They were subjected to CBCT scans, examined for alveolar process thickness (APT), facial bone thickness (FBT), facial bone thickness at the apex (FBTA) in mm and Tooth inclination in degrees in the maxillary anterior teeth including central incisors (CI), lateral incisors (LI) and canines.

**Results:**

The mean values for APT and FBTA for all teeth groups were found to be greater in males, while there was no gender difference between the FBT values. FBTA values were significantly greater for CI and LI in males. A strong inverse relationship was observed in males for tooth inclination with FBTA for all teeth, while in females, it was observed only for tooth inclination with FBTA for CI (*p *˂ 0.05).

**Conclusions:**

This study demonstrates distinct gender and tooth‐specific variations in APT, FBTA, and tooth inclination across the maxillary anterior region. Among all evaluated parameters, FBTA was identified as the sole alveolar bone feature independently associated with tooth inclination after adjusting for covariates and within‐subject clustering. Consequently, these findings offer vital anatomical and radiographic insights to aid treatment planning and enhance clinical outcomes in anterior maxillary procedures.

Trial Registration: CTRI number: CTRI/2022/06/055457; Registered with Clinical Trial Registry India on 04/07/2022.

## 1 Introduction

Dental implants have gained universal acceptance in the past few years, especially in cases requiring restoration of the maxillary anterior teeth. Alveolar process thickness (APT), its height, and the teeth angulation are pivotal components for the selection of various treatment strategies, particularly in the anterior maxillary region. After the removal of a tooth, the jawbone naturally undergoes changes. The bone tends to shrink and reshape itself, especially on the front and cheek sides, because the outer bone layer in these areas is very thin (Caiazzo et al. 2013). Significant alterations in bone dimensions occur in the buccal bone plate, with the palatal being a bit less affected aspect. Bone loss is usually greater in the front part of the upper jaw than in the back, because the bone plates there are naturally thinner and more prone to resorption. Subsequently, less intense remodeling of bone continues to follow up to about 12 months after extraction, contributing to the dimensional changes (Morjaria et al. 2014; Saldanha et al. 2006; Schropp et al. 2003). Many clinicians practice implant placement with a flapless approach as it preserves the stability of the underlying tissues. This procedure often needs to position the implant closer to the palatal side. However, if the bone in this area is insufficient, there is a risk of perforating the cortical plates, which can lead to implant failure.

The extent of bone resorption largely relies on the original thickness of the cortical bone on the buccal aspect. Thinner buccal plates are more prone to resorption, which can negatively impact the final esthetic outcome (Januário et al. 2011; Tomasi et al. 2010). Additionally, the amount of resorption of bone also depends on the angle between the axial inclination and the long axis of the tooth of the surrounding alveolar bone (Misawa et al. 2016).

Cone beam computed tomography (CBCT) images present a view that was three‐dimensional of all the anatomic structures that are in proximity to teeth and also in the sites that are edentulous, thereby providing the clinician with absolute clarity in the area of interest. A few preliminary studies have determined the alveolar bone dimensions previously using CBCT and indicated it as an aid in prognosis in predicting pre‐surgical and post‐surgical bone deficiencies in the treatment of implant (Zhang et al. 2015; Zhixuan et al. 2014).

On screening through the literature, it was realized that there is a paucity of information and understanding on the combined influence of alveolar process dimensions and teeth inclinations, especially of the individual teeth. Subtle differences between ethnicity, population characteristics, and gender have also been thought to have an impact on the thickness and height of the alveolar bone on the facial aspect. Additionally, it has been observed that the soft and hard tissue dimensions exhibit variability depending on the periodontal phenotype, with females predominantly demonstrating thinner dimensions, which naturally are prone to greater resorption (Kolte et al. 2023; Yadav et al. 2018). Recent evidence by Zhang et al. (2025) has highlighted that alveolar bone thickness and tooth inclination are significantly influenced by specific anterior arch forms, particularly in patients with skeletal Class II malocclusions. While their work emphasizes malocclusion‐driven variations, there remains a need to establish baseline age and gender variations in a broader, non‐orthodontic population to guide immediate implant protocols. Firincioglulari et al. (2024) suggest that bone dimensions are not only influenced by dental arch position but also exhibit significant variations pertaining to gender, a factor that must be accounted for during the planning phase of immediate implant therapy. So, such an evaluation and analysis were thought to be imperative to enable the clinician in appropriate treatment planning.

While several studies have utilized CBCT to evaluate the alveolar bone morphology of the anterior maxilla, there remains a lack of consensus regarding the extent of age and gender variations in these dimensions, particularly within the Indian population. Most existing literature focuses broadly on bone height and thickness without concurrently evaluating the relationship between APT, facial bone thickness at the apex (FBTA), and axial tooth inclination. Furthermore, there is a paucity of data regarding how anatomical variations might necessitate modified clinical protocols for immediate implant placement. This study seeks to bridge this gap by providing a comprehensive, gender‐based morphometric analysis of the maxillary anterior region.

Based on the identified gaps in the literature, the following hypothesis was formulated:

“There is a significant age and gender difference between APT and different tooth inclination patterns in the maxillary anterior region.” Hence, the current study was designed for the evaluation of variation in age and gender in the dimensions of the alveolar process and the inclinations of teeth in the maxillary anterior region using CBCT.

## 2 Methods

In this cross‐sectional study, 347 teeth from 60 periodontally healthy patients, equally comprising both genders in an age group of 20 to 60 years, were enrolled from the Department of Periodontics and Implantology. The CBCT scans used in this study were originally performed for various clinical indications, including the assessment of impacted teeth, orthodontic treatment planning, and preoperative evaluation for minor oral surgical procedures. Only scans that clearly visualized the maxillary anterior region and met the pre‐defined inclusion criteria were selected for analysis from the Department of Oral Medicine and Radiology at the Dental College and Hospital. The study adheres to STROBE guidelines. The complete outline with study details was explained to the patients, and informed consent was obtained in written format. The protocol was presented to the Institutional Ethics Committee of our institute, which granted the needful approval after being convinced that it adhered to the guidelines of the Helsinki Declaration of 1975, as revised in 2013. It was also registered with the Clinical Trials Registry of India, being the primary repository of the World Health Organization clinical trials platform.

### 2.1 Sample Size

Sample size estimation was done using the G Power version 3.1.9.2 software. The effect size of 0.31 was considered with alpha error, that is, 0.05% and 80% as the power of the study. A convenience sampling strategy was employed to identify suitable CBCT scans. All scans were evaluated against the inclusion and exclusion criteria by two independent, calibrated examiners. To ensure a balanced gender distribution, a stratified approach was used to include an equal number of male (*n* = 30) and female (*n* = 30) participants who met the clinical and radiographic health requirements.

The participants were involved in the study on fulfilling specified criteria, which are listed. The inclusion criteria were (a) Males and females between the age group of 20‐60 years, (b) Periodontal health as evaluated by Plaque index (PI) (Silness and Löe 1964) and Gingival index (GI) (Löe and Silness 1963) scores of 0 to 1, (c) Patients with fully erupted well‐aligned teeth without any orthodontic anomaly, (d) Systemically healthy patients without any medication intake on a regular basis, (e) Availability of good quality of the CBCT images, and (f) Teeth without any restorations. The exclusion criteria were (a) Artificial crowns, fillings, attrition on the teeth present in the maxillary anterior teeth, (b) Congenitally missing tooth or teeth, (c) Scans showing any signs of pulpal disease or periapical infections such as periapical radiolucency or root canal fillings in teeth next to the missing tooth area were excluded from the study.

### 2.2 Radiographic Analysis

Prior to performing linear and angular measurements, each CBCT volume was standardized using a fixed orientation protocol. The horizontal reference plane was oriented parallel to the hard palate (palatal plane). The vertical reference plane was aligned with the long axis of each individual tooth in both the sagittal and coronal sections. The CBCT scans for all the study participants were obtained with field of view (FOV) of 6 × 8 cm for 20 s through mandated protocol: 14‐bit grayscale; focal spot of 0.5 mm; 0.3 mm voxel size; single 360° rotation and amorphous silicon flat panel image detector. The images were obtained in DICOM (Digital Imaging and Communications in Medicine) format and later processed on a computer system. For each tooth, the parasagittal reconstructions were procured at the center of the socket. The measurements were recorded for maxillary central incisors (CI), lateral incisors (LI), and canines.

The clinical and radiographic examinations and recording of study parameters were done by a clinician who was calibrated prior to commencement of the study. The intra‐observer error was determined on ten scans that were randomly selected, and the variables were measured at two time points within 24‐h interval. Intraclass correlation coefficients (ICCs) were calculated separately for APT, facial bone thickness (FBT), FBTA, and tooth inclination (TI). The ICCs ranged from 0.82 to 0.87 across the four measurements, indicating good to excellent measurement reliability. The following parameters were recorded.

1. **APT:** APT was measured parallel to the axial plane as the distance extending from the facial boundary of the alveolar process to the palatal, located 3 mm apical to CEJ (Figure 1a,b).
2. **FBT:** To evaluate FBT, a line was drawn inside the facial bone wall, running parallel to the coronal margin of the alveolar process, at a level 3 mm apical to the CEJ (Figure 2a,b).
3. **FBTA:** FBTA was measured as the distance of the line running parallel to the axial plane, extending from the most apical point to the facial bone wall (Figure 2a,b).
4. **Tooth inclination:** It was defined as the angle formed by the tooth's long axis in relation to the axial plane of the skull (Figure 3a,b).

**Figure 1:**
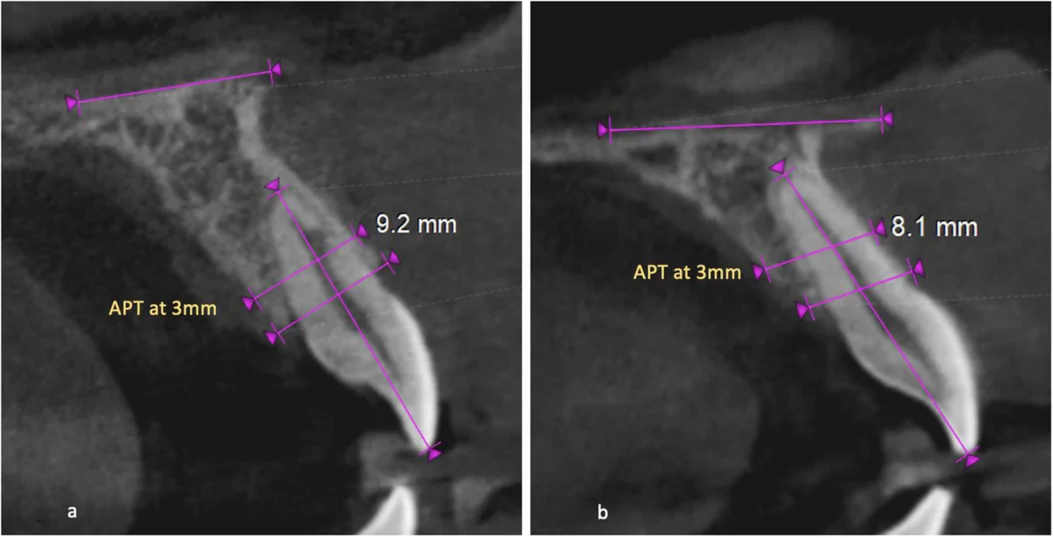
(a) Alveolar process thickness (APT) at 3 mm measurement in males. (b) APT at 3 mm measurement in females.

**Figure 2:**
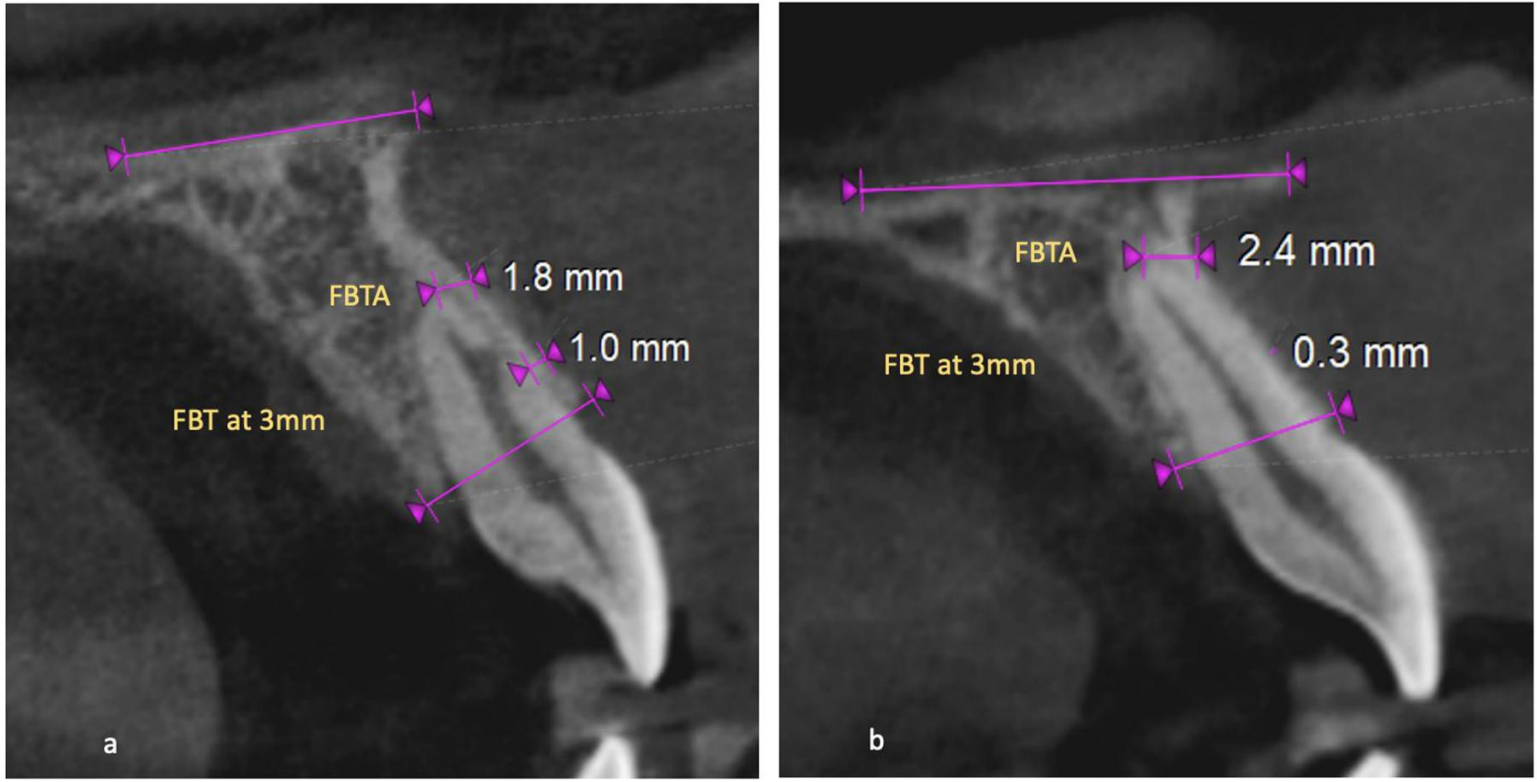
(a) Facial bone thickness (FBT) and facial bone thickness at the apex (FBTA) at 3 mm in males. (b) FBT and FBTA at 3 mm in females.

**Figure 3:**
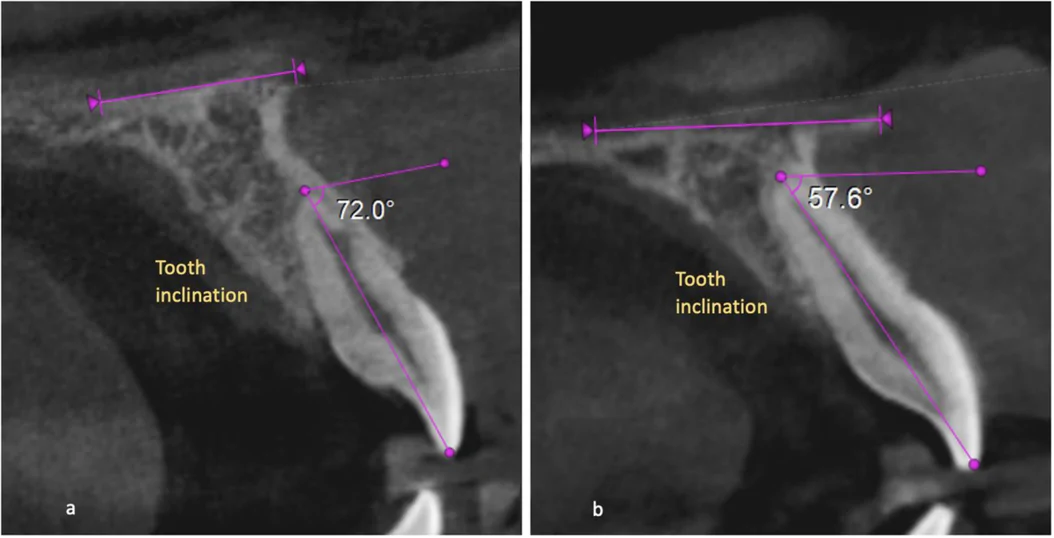
(a) Tooth inclination in males. (b) Tooth inclination in females.

The primary outcome variables were the determination of APT, FBT, FBTA, and tooth inclination, while the secondary outcome variables included gender wise correlation between the parameters.

### 2.3 Statistical Analysis

Statistical analyses were performed using R software (ver. 4.4.0; R Foundation for Statistical Computing, Vienna, Austria) and the statistical significance was evaluated at 5% level (two‐sided).

Continuous variables were summarized in terms of mean, standard deviation (SD), median, minimum, and maximum values as per tooth type and side, while categorical variables were summarized as frequencies and percentages.

Since each participant had measurements from multiple teeth, observations were not statistically independent. Therefore, the primary analysis was performed using a linear mixed‐effects model, with teeth (level 1) nested within participants (level 2). Tooth inclination (TI) was considered the dependent variable, while APT, FBT, FBTA, age, gender, and tooth position were included as fixed effects. A participant‐specific random intercept was incorporated to account for the within‐subject correlation arising from repeated measurements on multiple teeth from the same participant. Gender differences in APT, FBT, FBTA, and TI were assessed using separate linear mixed‐effects models to account for the clustering of multiple tooth‐level observations within participants. Adjusted marginal means for males and females and the corresponding differences were estimated using the estimated marginal means method, with 95% confidence intervals and two‐sided *p* values. Associations between tooth inclination and APT, FBT, and FBTA were evaluated using separate linear mixed‐effects models, with the respective alveolar bone parameter, gender, and tooth type as fixed effects and participant as a random intercept to account for clustering of multiple teeth within participants. Age, gender, and tooth‐type‐specific regression coefficients (β) with 95% confidence intervals and two‐sided *p* values were obtained using estimated marginal trends. These analyses were considered secondary/exploratory.

## 3 Results

Altogether 60 CBCT scans comprising equal numbers of male and female participants (Cumulatively, 347 teeth were evaluated, specifically 176 from males (60 CI, 60 LI, and 56 canines) and 171 from females (58 CI, 59 LI, and 54 canines)) were analyzed. The overall mean age of the participants 38.22 ± 10.87 years, with a median of 34.5 years. The minimum age was 22 years, while the maximum was 60 years. There were 30 (50%) males and 30 (50%) females in the study. The mean age of males was 38.57 ± 11.02 years, with a median of 35 years, while the mean age of females was 37.87 ± 10.73 years, with a median age of 34.50 years (Table 1).

**Table 1:** Demographic details of the participants.

Characteristic	*n*	Mean ± SD; median	Minimum	Maximum
Age in years (overall)	60	38.22 ± 10.87; 34.50	22	60
Gender				
Male	30 (50%)	38.57 ± 11.02; 35.00	22	60
Female	30 (50%)	37.87 ± 10.73; 34.50	22	57

Table 2 shows that gender‐related differences varied according to the tooth type and parameter examined. Among CI, males had significantly greater adjusted mean FBTA than females (mean difference, −0.53 mm; 95% CI, −0.96 to −0.09; *p* = 0.018), whereas no significant gender differences were observed for APT, FBT, or TI.

**Table 2:** Adjusted comparison of alveolar parameters and tooth inclination between males and females according to tooth type.

Tooth type	Parameter	Adjusted mean (95% CI)		Mean difference (95% CI)	*p* value
		Male	Female		
Central incisors	APT (mm)	9.26 (8.79, 9.73)	8.88 (8.41, 9.35)	−0.38 (−1.04, 0.29)	0.259
	FBT (mm)	0.78 (0.69, 0.86)	0.79 (0.70, 0.87)	0.11 (−0.11, 0.13)	0.856
	FBTA (mm)	2.58 (2.28, 2.89)	2.05 (1.75, 2.36)	−0.53 (−0.96, −0.09)	**0.018**
	TI (degree)	68.9 (64.1, 73.7)	72.1 (67.3, 76.9)	3.21 (−3.56, 9.98)	0.346
Lateral incisor	APT (mm)	8.35 (7.93, 8.76)	7.72 (7.30, 8.14)	−0.62 (−1.21, −0.03)	**0.041**
	FBT (mm)	0.64 (0.58, 0.70)	0.65 (0.59, 0.72)	0.01 (−0.08, 0.24)	0.809
	FBTA (mm)	2.33 (2.06, 2.60)	1.91 (1.64, 2.18)	−0.41 (−0.79, −0.03)	**0.034**
	TI (degree)	78.1 (74.4, 81.7)	79.3 (75.7, 82.9)	1.26 (−3.85, 6.37)	0.623
Canines	APT (mm)	10.0 (9.51, 10.5)	8.90 (8.40, 9.40)	−1.11 (−1.81, −0.41)	**0.002**
	FBT (mm)	0.71 (0.49, 0.94)	0.74 (0.51, 0.97)	0.03 (−0.29, 0.35)	0.863
	FBTA (mm)	2.30 (2.00, 2.60)	2.00 (1.71, 2.30)	−0.29 (−0.72, 0.12)	0.158
	TI (degree)	89.7 (86.7, 92.8)	90.4 (87.4, 93.5)	0.71 (−3.62, 5.03)	0.745

*Note:* Mean differences represent female − male. Adjusted means and 95% confidence intervals were estimated from linear mixed‐effects models accounting for clustering of each type of teeth within participants; Bold *p* values indicate statistical significance.

Among LI, males had significantly greater adjusted mean APT than females (mean difference, −0.62 mm; 95% CI, −1.21 to −0.03; *p* = 0.041). Further, the adjusted mean FBTA in males was higher than in females (mean difference, −0.41 mm; 95% CI, −0.79 to −0.03; *p* = 0.034). No significant differences were observed for FBT or TI.

Among canines, males had significantly greater adjusted mean APT than females (mean difference, −1.11 mm; 95% CI, −1.81 to −0.41; *p* = 0.002). However, other parameters did not differ significantly between males and females.

Table 3 shows that the primary multivariable linear mixed‐effects model was used to evaluate the association of tooth inclination (TI) with APT, FBT, and FBTA, while accounting for the clustering of teeth within each participant and adjusting for age, gender, and tooth position. After adjustment for covariates, FBTA was the only parameter significantly associated with tooth inclination. In other words, each 1‐mm increase in FBTA was associated with a 3.77° decrease in TI (β = −3.77, SE = 0.72, *p* < 0.001). In contrast, neither APT (β = 0.47, SE = 0.44, *p* = 0.284) nor FBT (β = −0.61, SE = 0.91, *p* = 0.498) showed a statistically significant independent association with TI.

**Table 3:** Multivariable linear mixed‐effects model of factors associated with tooth inclination.

Factor	β	SE	95% CI	Std. β (95% CI)	*p* value
Age (years)	−0.07	0.1	−0.27, 0.13	−0.06 (−0.21, 0.10)	0.477
Gender: Male	−0.31	2.22	−4.67, 4.04	−0.02 (−0.33, 0.29)	0.889
APT (mm)	0.47	6.06	−0.39, 1.33	0.05 (−0.04, 0.15)	0.284
FBT (mm)	−0.61	0.91	−2.39, 1.16	−0.02 (−0.09, 0.04)	0.498
FBTA (mm)	−3.77	0.72	−5.18, −2.36	−0.24 (−0.33, −0.15)	**< 0.001**
LI (Right)	8.43	1.48	5.53, 11.32	0.60 (0.39, 0.80)	**< 0.001**
C (Right)	17.05	1.44	14.23, 19.88	1.21 (1.01, 1.41)	**< 0.001**
CI (Left)	0.23	1.42	−2.56, 3.01	0.02 (−0.18, 0.21)	0.874
LI (Left)	7.41	1.50	4.47, 10.34	0.52 (0.32, 0.73)	**< 0.001**
C (Left)	20.88	1.47	18.00, 23.76	1.48 (1.27, 1.68)	**< 0.001**

*Note:* Linear mixed‐effects model with individual‐specific random intercept. Fixed effects include age, gender, APT, FBT, FBTA, and tooth position; Reference levels: Female for gender and right central incisor for tooth position. Bold *p* values indicate statistical significance.

Tooth position was a significant predictor of TI. Compared with the right central incisor (reference category), the lateral incisor (β = 8.43, *p* < 0.001), the right canine (β = 17.05, *p* < 0.001), the left lateral incisor (β = 7.41, *p* < 0.001), and the left canine (β = 20.88, *p* < 0.001) exhibited significantly greater tooth inclination.

A linear mixed‐effects model with a participant‐specific random intercept was fitted to evaluate the association of tooth inclination (TI) with APT, FBT, and FBTA, while accounting for the clustering of six teeth within each participant and adjusting for age, gender, and tooth position.

After adjustment for covariates, FBTA was the only parameter significantly associated with tooth inclination. In other words, each 1‐mm increase in FBTA was associated with a 3.77° decrease in TI (β = −3.77, SE = 0.72, *p* < 0.001). In contrast, neither APT (β = 0.47, SE = 0.44, *p* = 0.284) nor FBT (β = −0.61, SE = 0.91, *p* = 0.498) showed a statistically significant independent association with TI.

Age (β = −0.07, SE = 0.10, *p* = 0.477) and gender (male vs. female: β = −0.31, SE = 2.22, *p* = 0.889) were also not significantly associated with tooth inclination. (Figure 4).

**Figure 4:**
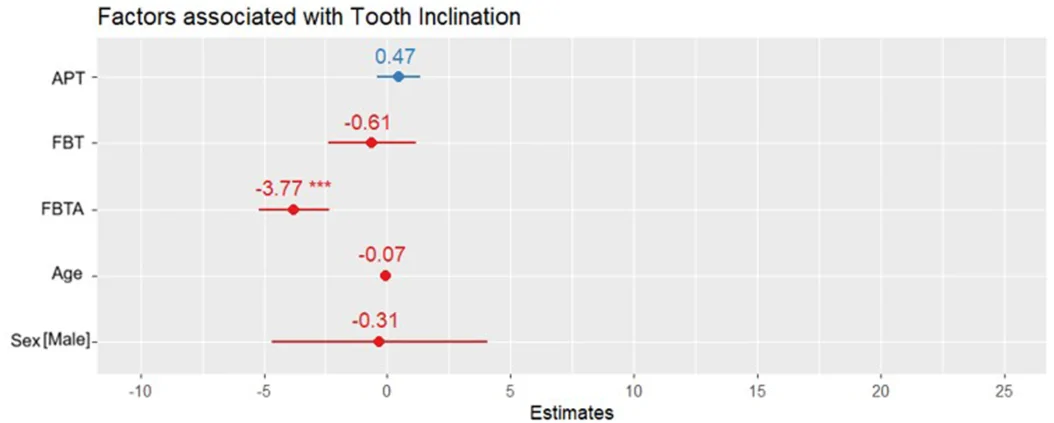
Forest plot of adjusted regression coefficients obtained from linear mixed‐effects model for factors associated with tooth inclination. ***p < 0.001.

The relationship between alveolar bone parameters and tooth inclination could potentially vary according to gender and tooth type, and therefore secondary mixed‐effects analyses were performed to examine these associations between males and females and tooth‐type strata, with results shown in Table 4.

**Table 4:** Gender‐ and tooth‐type‐specific associations between alveolar bone parameters and tooth inclination: secondary mixed‐effects analysis.

Tooth type	Gender	Alveolar bone parameter [β (95% CI); *p* value]		
		APT	FBT	FBTA
Central incisors	Male	−0.05 (−1.79, 1.68); 0.952	−10.51 (−19.02, −2.00); **0.016**	−6.51 (−9.31, −3.71); **< 0.001**
	Female	0.79 (−1.15 2.74); 0.424	0.17 (−8.69, 9.04); 0.969	−4.07 (−6.49, −1.65); **0.001**
Lateral incisor	Male	0.74 (−1.06, 2.53); 0.420	−1.53 (−10.19, 7.13); 0.728	−5.32 (−8.13, −2.50); **< 0.001**
	Female	−0.64 (−3.03, 1.76); 0.623	−3.22 (−12.24, 5.79); 0.482	0.08 (−2.91, 3.07); 0.957
Canines	Male	2.00 (0.26, 3.74); **0.025**	−2.58 (−9.36, 4.21); 0.456	−4.11 (−6.70, −1.52); **0.002**
	Female	−0.26 (−1.76, 1.24); 0.735	0.18 (−1.89, 2.26); 0.862	−0.49 (−3.92, 2.95); 0.781

*Note:* β coefficients represent the adjusted change in tooth inclination (degrees) per unit increase in the respective alveolar bone parameter. Models accounted for clustering of multiple teeth within participants using a participant‐level random intercept. Bold *p* values indicate statistical significance.

Among male CI, FBT was significantly negatively associated with TI (β = −10.51°, 95% CI, −19.02 to −2.00; *p* = 0.016), indicating that each 1‐mm increase in FBT was associated with an estimated 10.5° decrease in TI. FBTA also showed a significant negative association with TI (β = −6.51°, 95% CI, −9.31 to −3.71; *p* < 0.001). APT was not significantly associated with TI.

Among female CI, FBTA was significantly negatively associated with TI (β = −4.07°, 95% CI, −6.49 to −1.65; *p* = 0.001), whereas APT and FBT were not significantly associated with TI.

For LI, FBTA was significantly negatively associated with TI among males (β = −5.32°, 95% CI, −8.13 to −2.50; *p* < 0.001), while APT and FBT were not significantly associated with TI. Among females, none of the three alveolar bone parameters showed a statistically significant association with TI.

For canines, APT was positively associated with TI among males (β = 2.00°, 95% CI, 0.26 to 3.74; *p* = 0.025), whereas FBTA was negatively associated with TI (β = −4.11°, 95% CI, −6.70 to −1.52; *p* = 0.002). FBT was not significantly associated with TI. Among female canines, none of the alveolar bone parameters was significantly associated with TI.

The conceptual difference between Tables 3 and 4 is that the former table estimates the overall independent association of APT, FBT, and FBTA with TI while simultaneously adjusting for age, gender, individual tooth position, and accounting for the participant‐level clustering. The latter table explores the relationship of TI with bone parameters according to gender and tooth type by accounting for patient‐level clustering.

## 4 Discussion

This study determines the APT, FBT, FBTA, and tooth inclination for individual teeth in males and females in the maxillary anterior sextant using CBCT scans. It is extremely important to know the dimensions of the bone in cases of immediate implant placement in the maxillary anterior sextant, as these have a direct bearing on the cumulative implant survival (Botticelli et al. 2008; Lang et al. 2012). The anterior maxilla also presents challenges for the clinician due to the demands of well‐anchored immediate implants without compromising on the esthetics. Multiple factors contribute to a higher risk of esthetic complications, such as the lack of bundle bone, remodeling of the alveolar process following extraction, thickness of the buccal bone wall, and the anatomical convexity of the alveolar ridge.

The results of the current study are indicative of the mean values of APT in both males and females for canines, which were the maximum, followed by CI, and then the LI. The position of LI within the arch coincides with the buccal concavity arising from the lateral fossa, which is reflected in the bone dimensions. These dimensional differences point towards the requirements of complete housing of the root surfaces in the alveolar process, and since the root size of maxillary canines is more than that of the other teeth types, so are the dimensions of APT. The literature does not document combined measurements of palatal and buccal process thickness (APT); however, some studies have focused on the cross‐sectional dimensions of the alveolar process (Botelho et al. 2020). The palatal bone thickness at 3 mm apical to CEJ is an important aspect that is often ignored by many clinicians; however, it guides the placement of implants. Our results, showing significantly greater APT in males for certain tooth types, are consistent with the data reported by Firincioglulari et al. (2024). In their study of 300 maxillary incisors, they observed that male participants consistently exhibited higher bone thickness values across multiple measurement points. This collective evidence reinforces the theory that gender is a significant biological determinant of the available bone housing in the anterior maxilla. The authors of the abovementioned study also identified a trend where alveolar bone thickness appears to decrease with advancing age. This highlights the importance of considering both gender and age‐related physiological remodeling when predicting the long‐term stability of the facial bone wall following tooth extraction.

In a few of the previously reported studies, the FBT at 3 mm apical to the CEJ was in the majority of the cases ˂ 1 mm (Kheur et al. 2015; Dos Santos et al. 2019). The outcomes of the current investigation are in accordance with the above observations, wherein the mean values of FBT did not exceed 1 mm and exhibited a presence of thin phenotype. To be more precise, the FBT values were found to be in a range of 0.10 to 1.7 mm. In one of the studies wherein the authors examined 1463 teeth, the FBT values were reported to be 0.1 to 1 mm (Dos Santos et al. 2019).

The FBTA measurements, which were recorded at the root apex, indicated that the males consistently demonstrated greater mean values for all the teeth types. The differences between the genders were found to be significant for CI and LI tooth types, while the values did not achieve statistical significance for the canines. The FBTA values in the present study were somewhat greater, being 2.58 ± 0.85 mm, 2.33 ± 0.83 mm, and 2.31 ± 0.97 mm in males, while those in females were 2.05 ± 1.00 mm, 1.92 ± 0.81 mm, and 2.05 ± 0.72 mm for CI, LI, and canines, respectively, as compared to the previous investigations by Botelho et al and Kheur et al. However, a former study has mentioned the mean values for all the maxillary anterior teeth together, which were recorded at 7 mm apically to the CEJ and not the apex, while the latter mentioned only for the CI. The present study findings, being individualized measurements for each tooth type, should be considered to be more specific and precise. Our results, indicating that the FBT across all anterior tooth types rarely exceeded 1 mm, align closely with the observations of Sağlıklı and İpek (2023). Their CBCT evaluation of the anterior maxillary region revealed that the buccal bone is characteristically thin, particularly at the crestal and mid‐root levels. This cross‐sectional study's consistency reinforces the necessity of a cautious surgical approach, regardless of the patient's gender, when the facial plate is inherently delicate.

In this study, tooth angulation was assessed by measuring the angle between the long axis of the tooth and the axial plane, following the method described by Botelho et al while some of the studies have used the adjacent contralateral angle formed with the long axis (Nahás‐Scocate et al. 2014) while some others have used angle formed between axis of the tooth and the axis of the basal bone triangle (López‐Jarana et al. 2018) and still others have used the angle formed by the long axis of the tooth with the long axis of the alveolar process (Dos Santos et al. 2019; Wang et al. 2014). The method adopted by the present study appears to be more relevant as the angulation has to be measured at the apex point of the tooth as it decides whether the tooth is inclined in relation to the alveolar bone. Also, it is the root and its three‐dimensional position which most of the times determines the exact alveolar housing area and thus aids in the selection of appropriate implant dimension. So accordingly, a significant negative correlation was observed for tooth inclination with FBT in CI, while for all the teeth, that is, CI, LI, and canines, it was in relation to FBTA in males. For females, this negative correlation was significant only with FBTA for CI, while that for other teeth and parameters, it did not achieve statistical significance. The above findings indicate that as the tooth angulation increases, there is a decrease in the FBTA and vice versa. A significant direct correlation with the thickness of the apical buccal region or CI was observed by Nahás‐Scocate et al. while an inverse correlation between the angle, maxillary central incisor axis, and the palatal plane was reported by Mirmohamadsadeghi et al. (2021).

Overall, APT and FBTA dimensions were greater in males, whereas the tooth angulation was greater in females. The observed variations in APT and FBTA between genders may be associated with adaptive bone remodeling in response to masticatory forces. Previous literature suggests that higher occlusal loads, often reported in male cohorts, could influence alveolar bone thickness; however, further longitudinal studies are required to confirm this relationship in the current population.

The mixed‐effects analysis highlighted that FBTA was the only alveolar bone parameter independently associated with tooth inclination after controlling for potential confounders and the correlation arising from multiple teeth being evaluated within the same participant. This finding suggests that the apical facial bone may have a greater influence on the spatial orientation of maxillary anterior teeth than the crestal facial bone or overall APT. The absence of independent associations for APT and FBT indicates that these measurements alone may not reliably predict tooth inclination when anatomical tooth position and within‐subject variability are considered. The significant effect of tooth position further supports the presence of intrinsic anatomical differences in inclination among the maxillary anterior teeth, whereas age and gender did not appear to influence tooth inclination in the present cohort.

Affendi et al. (2023) demonstrated a significant association between apical facial bone thickness and tooth angulation, whereas the crestal facial bone was predominantly thin and less consistently related to tooth angulation. This provides direct support for your finding that FBTA, rather than FBT, is associated with tooth inclination.

The gender‐specific analyses demonstrated differences in alveolar bone parameters between males and females, with males exhibiting significantly greater FBTA in the CI and LI, as well as greater APT in the LI and canines. Furthermore, the unadjusted correlation analysis showed significant inverse correlations between tooth inclination and FBTA across all maxillary anterior teeth in males, whereas these associations were less consistent in females. However, these findings should be interpreted with caution, as they do not account for the correlation arising from multiple teeth being evaluated within the same participant or for other potential confounding factors. After applying a linear mixed‐effects model that accounted for within‐subject clustering and adjusted for age, gender, and tooth position, FBTA remained the only independent predictor of tooth inclination, while neither age nor gender showed a significant association. This suggests that the observed gender specific differences in the unadjusted analyses were largely explained by anatomical tooth position and within‐subject variability.

The present study has several limitations. Its cross‐sectional design precludes causal inference, and the relatively small sample size and single ethnic cohort may limit the generalizability of the findings. The use of convenience sampling from CBCT scans obtained for various clinical indications may have introduced selection bias. Although the analyses were adjusted for age, gender, and tooth position, other potential confounders, including arch form, occlusion, and skeletal or dental patterns, were not evaluated. Furthermore, because multiple teeth were assessed within each participant, the data had an inherently clustered structure, although this was accounted for using a linear mixed‐effects model. Future longitudinal research is desirable to confirm these findings.

## 5 Conclusions

This study demonstrated gender and tooth‐specific variations in APT, FBTA, and tooth inclination in the maxillary anterior region. After accounting for within‐subject clustering and relevant covariates, FBTA emerged as the only alveolar bone parameter independently associated with tooth inclination. These findings provide valuable anatomical and radiographic insights into the relationship between tooth inclination and alveolar bone morphology. The observed anatomical variations may assist clinicians in the radiographic assessment and treatment planning of immediate implant placement in the maxillary anterior region. While these findings offer critical baseline radiographic data for pre‐surgical risk assessment, prospective longitudinal studies evaluating post‐extraction remodeling are needed to determine their direct impact on clinical implant outcomes.

## Author Contributions

R.K., A.K., and V.B. have made substantial contributions to conception and design, or acquisition of data, or analysis and interpretation of data. R.K., A.K., V.B., and R.G. have been involved in drafting the manuscript or revising it critically for important intellectual content. R.K., A.K., V.B., R.G., and G.D. have given final approval of the version to be published. Each author should have participated sufficiently in the work to take public responsibility for appropriate portions of the content. R.K., A.K., and G.D. agreed to be accountable for all aspects of the work in ensuring that questions related to the accuracy or integrity of any part of the work are appropriately investigated and resolved.

## Funding

The authors have nothing to report.

## Conflicts of Interest

The authors declare no conflicts of interest.

## Data Availability

The data that support the findings of this study are available from the corresponding author upon reasonable request.

## References

[cre270508-bib-0001] Affendi, N. , J. Babiker , and M. Mohd Yusof . 2023. “CBCT Assessment of Alveolar Bone Wall Morphology and Its Correlation With Tooth Angulation in the Anterior Mandible: A New Classification for Immediate Implant Placement.” Journal of Periodontal & Implant Science 53, no. 6: 453–466. 10.5051/jpis.2105000250 PMC10761280 37038832

[cre270508-bib-0002] Botelho, S. V. , J. Perussolo , M. Y. Misawa , H. H. Zadeh , and M. G. Araújo . 2020. “The Basal Bone and Alveolar Process in the Maxillary Anterior Region in Humans: A Cone Beam Computed Tomographic Study.” International journal of periodontics & restorative dentistry 40: 907–914. 10.11607/prd.4571 33151198

[cre270508-bib-0003] Botticelli, D. , A. Renzi , J. Lindhe , and T. Berglundh . 2008. “Implants in Fresh Extraction Sockets: A Prospective 5‐year Follow‐Up Clinical Study.” Clinical Oral Implants Research 19: 1226–1232. 10.1111/j.1600-0501.2008.01620.x 19040437

[cre270508-bib-0004] Caiazzo, A. , F. Brugnami , and P. Mehra . 2013. “Buccal Plate Preservation With Immediate Post‐Extraction Implant Placement and Provisionalization: Preliminary Results of a New Technique.” International Journal of Oral and Maxillofacial Surgery 42: 666–670. 10.1016/j.ijom.2012.11.009 23219710

[cre270508-bib-0005] Firincioglulari, M. , S. Koral , D. Kurt , and K. Orhan . 2024. “Gender‐Based Variation in Alveolar Bone Thickness of Maxillary Incisor Teeth: A CBCT Retrospective Study.” Medical Science Monitor 30: e944588. 10.12659/MSM.944588 PMC11302216 39001576

[cre270508-bib-0006] Januário, A. L. , W. R. Duarte , M. Barriviera , J. C. Mesti , M. G. Araújo , and J. Lindhe . 2011. “Dimension of the Facial Bone Wall in the Anterior Maxilla: A Cone‐Beam Computed Tomography Study.” Clinical Oral Implants Research 22: 1168–1171. 10.1111/j.1600-0501.2010.02086.x 21320168

[cre270508-bib-0007] Kheur, M. G. , N. R. Kantharia , S. M. Kheur , A. Acharya , and B. Le . 2015. “Three‐Dimensional Evaluation of Alveolar Bone and Soft Tissue Dimensions of Maxillary Central Incisors for Immediate Implant Placement: A Cone‐Beam Computed Tomography Assisted Analysis.” Implant Dentistry Publish Ahead of Print: 407–415. 10.1097/ID.0000000000000259 25930095

[cre270508-bib-0008] Kolte, R. A. , A. P. Kolte , and A. R. Purohit . 2023. “Gender‐Based Evaluation of Interproximal Dimensions: Influence on Interdental Papillae and Gingival Thickness in Maxillary Anterior Teeth.” International Journal of Esthetic Dentistry 18: 128–140. 37166768

[cre270508-bib-0009] Lang, N. P. , L. Pun , K. Y. Lau , K. Y. Li , and M. C. Wong . 2012. “A Systematic Review on Survival and Success Rates of Implants Placed Immediately Into Fresh Extraction Sockets After at Least 1 Year.” Clinical Oral Implants Research 23, no. Suppl. 5: 39–66. 10.1111/j.1600-0501.2011.02372.x 22211305

[cre270508-bib-0010] Löe, H. , and J. Silness . 1963. “Periodontal Disease in Pregnancy. I. Prevalence and Severity.” Acta Odontologica Scandinavica 21: 533–551. 10.3109/00016356309011240 14121956

[cre270508-bib-0011] López‐Jarana, P. , C. M. Díaz‐Castro , A. Falcão , C. Falcão , J. V. Ríos‐Santos , and M. Herrero‐Climent . 2018. “Thickness of the Buccal Bone Wall and Root Angulation in the Maxilla and Mandible: An Approach to Cone Beam Computed Tomography.” BMC Oral health 18: 194. 10.1186/s12903-018-0652-x PMC6249849 30463614

[cre270508-bib-0012] Mirmohamadsadeghi, H. , R. Morvaridi Farimani , F. Alzwghaibi Aws , S. Tohidkhah , and H. N. Alzamili . 2021. “Effect of Maxillary Central Incisor Inclination on Palatal Bone Width.” Journal of Dental School 39, no. 2: 48–53.

[cre270508-bib-0013] Misawa, M. , J. Lindhe , and M. G. Araújo . 2016. “The Alveolar Process Following Single‐Tooth Extraction: A Study of Maxillary Incisor and Premolar Sites in Man.” Clinical Oral Implants Research 27: 884–889. 10.1111/clr.12710 26566965

[cre270508-bib-0014] Morjaria, K. R. , R. Wilson , and R. M. Palmer . 2014. “Bone Healing After Tooth Extraction With or Without an Intervention: A Systematic Review of Randomized Controlled Trials.” Clinical Implant Dentistry and Related Research 16: 1–20. 10.1111/j.1708-8208.2012.00450.x 22405099

[cre270508-bib-0015] Nahás‐Scocate, A. C. R. , A. de Siqueira Brandão , M. P. Patel , M. E. Lipiec‐Ximenez , I. Chilvarquer , and K. M. Do valle‐Corotti . 2014. “Bone Tissue Amount Related to Upper Incisors Inclination.” Angle Orthodontist 84: 279–285.wa. 10.2319/031213-211.1 PMC8673802 23883305

[cre270508-bib-0016] Sağlıklı, A. , and F. İpek . 2023. “Evaluation of the Buccal Bone Thickness in the Anterior Maxillary Region Using Cone‐Beam Computed Tomography.” International Dental Research 13: 1–10.

[cre270508-bib-0017] Saldanha, J. B. , M. Z. Casati , F. H. Neto , E. A. Sallum , and F. H. Nociti, Jr. 2006. “Smoking May Affect the Alveolar Process Dimensions and Radiographic Bone Density in Maxillary Extraction Sites: A Prospective Study in Humans.” Journal of Oral and Maxillofacial Surgery 64: 1359–1365. 10.1016/j.joms.2006.05.021 16916669

[cre270508-bib-0018] Dos Santos, J. G. , A. P. O. R. Durão , A. C. C. Felino , and R. M. L. F. Almeida . 2019. “Analysis of the Buccal Bone Plate, Root Inclination and Alveolar Bone Dimensions in the Jawbone: A Descriptive Study Using Cone‐Beam Computed Tomography.” Journal of Oral & Maxillofacial Research 10: e4. 10.5037/jomr.2019.10204 PMC6683387 31404187

[cre270508-bib-0019] Schropp, L. , A. Wenzel , L. Kostopoulos , and T. Karring . 2003. “Bone Healing and Soft Tissue Contour Changes Following Single‐Tooth Extraction: A Clinical and Radiographic 12‐Month Prospective Study.” International Journal of Periodontics & Restorative Dentistry 23: 313–323. 12956475

[cre270508-bib-0020] Silness, J. , and H. Löe . 1964. “Periodontal Disease in Pregnancy. II. Correlation Between Oral Hygiene and Periodontal Condition.” Acta Odontologica Scandinavica 22: 121–135. 10.3109/00016356408993968 14158464

[cre270508-bib-0021] Tomasi, C. , M. Sanz , D. Cecchinato , et al. 2010. “Bone Dimensional Variations at Implants Placed in Fresh Extraction Sockets: A Multilevel Multivariate Analysis.” Clinical Oral Implants Research 21: 30–36. 10.1111/j.1600-0501.2009.01848.x 20070744

[cre270508-bib-0022] Wang, H. M. , J. W. Shen , M. F. Yu , X. Y. Chen , Q. H. Jiang , and F. M. He . 2014. “Analysis of Facial Bone Wall Dimensions and Sagittal Root Position in the Maxillary Esthetic Zone: A Retrospective Study Using Cone Beam Computed Tomography.” International Journal of Oral & Maxillofacial Implants 29: 1123–1129. 10.11607/jomi.3348 25216138

[cre270508-bib-0023] Yadav, S. , E. Sachs , M. Vishwanath , et al. 2018. “Gender and Growth Variation in Palatal Bone Thickness and Density for Mini‐Implant Placement.” Progress in Orthodontics 19: 43. 10.1186/s40510-018-0241-1 PMC6215790 30393829

[cre270508-bib-0024] Zhang, W. , A. Skrypczak , and R. Weltman . 2015. “Anterior Maxilla Alveolar Ridge Dimension and Morphology Measurement by Cone Beam Computerized Tomography (CBCT) for Immediate Implant Treatment Planning.” BMC Oral health 15: 65. 10.1186/s12903-015-0055-1 PMC4460662 26059796

[cre270508-bib-0025] Zhang, Y. , J. Wang , Y. Yin , et al. 2025. “Anterior Arch Form‐Specific Analysis of Alveolar Bone Thickness and Tooth Inclination: Associations and Clinical Relevance in Skeletal Class II Malocclusion.” BMC Oral Health 25: 1783. 10.1186/s12903-025-07121-9 PMC12619305 41239390

[cre270508-bib-0026] Zhixuan, Z. , C. Wu , S. Ming , S. Chao , L. Jun , and C. Ning . 2014. “Cone Beam Computed Tomographic Analyses of Alveolar Bone Anatomy at the Maxillary Anterior Region in Chinese Adults.” Journal of Biomedical Research 28: 498–505. 10.7555/JBR.27.20130002 PMC4250963 25469120

